# Effect of a rigid ankle foot orthosis and an ankle foot orthosis with an oil damper plantar flexion resistance on pelvic and thoracic movements of patients with stroke during gait

**DOI:** 10.1186/s12938-023-01068-0

**Published:** 2023-02-06

**Authors:** Hua Ling, Hui Guo, Hao Zhou, Xiao-Qian Chang, Zi-Yang Guo, Sumiko Yamamoto, Li-Fei Cai, Jun Zhao

**Affiliations:** 1 Rehabilitation Engineering Institute, China Rehabilitation Science Institute, No.18, Jiaomen Beilu, Beijing, 100068 China; 2 Beijing Bo’ai Hospital, China Rehabilitation Research Center, No.10, Jiaomen Beilu, Beijing, 100068 China; 3 Graduate School, International University of Health & Welfare, 4‑1‑26 Akasaka, Minato‑ku, Tokyo, 107‑8402 Japan

**Keywords:** Ankle–foot orthosis, Stroke, Trunk movement, Gait, Rehabilitation, Biomechanics

## Abstract

**Background:**

Impairments of trunk movements in gait of stroke are often reported. Ankle foot orthosis (AFO) is commonly used to improve gait of stroke; however, the effect of different types of AFOs on the pelvic and thoracic movements during gait in stroke has not been clarified.

**Methods:**

Thirty-four patients with stroke were randomly allocated to undergo 2 weeks of gait training by physiotherapists while wearing a rigid AFO (RAFO) with a fixed ankle or an AFO with an oil damper (AFO-OD) that provides plantarflexion resistance and free dorsiflexion. A motion capture system was used for measurements of shod gait without AFO at baseline and with and without AFO after gait training. Two-way repeated ANOVA, Wilcoxon signed-rank test, and Mann–Whitney *U* test were performed for the data after the gait training to know the effect of different kinds of AFOs.

**Results:**

Twenty-nine patients completed the study (AFO-OD group: 14, RAFO group: 15). Interactions were found in pelvic rotation angle, change of shank-to-vertical angle (SVA) in the stance, and paretic to non-paretic step length, which increased in AFO-OD group with AFOs (*p* < 0.05), while the SVA decreased in RAFO group with AFOs (*p* < 0.05). The main effects were found in pelvic rotation at the contralateral foot off, and thoracic tilt at foot off when an AFO was worn. The change of SVA in stance was positively correlated with the pelvic rotation in the AFO-OD group (*r* = 0.558). At initial contact, pelvic rotation was positively correlated with thoracic rotation in both groups.

**Conclusions:**

The findings in 29 patients with stroke showed that pelvic and thoracic movements especially the rotation were affected by the type of AFOs. Pelvic rotation and lower limb kinematics exhibited significant improvements with AFO-OD, reflecting more desirable gait performance. On the other hand, the increase in thoracic in-phase rotation might expose the effect of insufficient trunk control and dissociation movement.

*Trial registration* UMIN000038694, Registered 21 November 2019, https://center6.umin.ac.jp/cgi-open-bin/ctr_e/ctr_his_list.cgi?recptno=R000044048.

**Supplementary Information:**

The online version contains supplementary material available at 10.1186/s12938-023-01068-0.

## Background

Stroke is the primary cause of mortality and disability for adults [1]. Stroke is an abrupt neurological outburst caused by cerebrovascular perfusion injury that may result in neuronal function impairment associated with motor, cognition, or sensation [2, 3]. Patients after stroke not only have unilateral hemiplegia but also have difficulties in the trunk control [4], affecting their balance, gait, and daily living activities [5–7].

Many studies found that variability of trunk movement increased significantly in the gait of stoke. The paretic pelvis was upward in the stance phase and downward in the swing phase, contrary to the healthy group [10, 11]. To ensure the foot clearance in the paretic swing phase [12], the trunk of the non-paretic side showed a larger lateral swing during the stance phase [13]. The pelvis showed excessive forward tilts during the gait cycle [14]. In addition, the accelerations of the thorax and pelvis were greater in patients with stroke than in controls. Moreover, the thorax has an excessive rotation, while the rotation of the pelvis was less than that of the thorax in the stroke gait [15]. A few studies focused on the synchronous relative phase difference in the thorax and pelvis. One study found significant differences in in-phase rotation of the thorax and pelvis, and there was more in-phase rotation of the trunk as the velocity decreased [15], while no significant result was found in another two studies [16, 17]. Kinematic parameters of the trunk movements were changed in the gait after stroke, which was manifested as excessive movements in the sagittal and coronal planes, reduced anti-phase rotation of the thorax and pelvis, affected symmetry, and increased the instability of the trunk. The trunk movement was an important part of gait retraining for patients with stroke [8, 9] and could be one of the predictors of the overall function of gait [5].

Moreover, the impairments of the trunk function could not only affect trunk movement but also affect lower limb motor recovery. Several studies illustrated that thoracic rotation and pelvic obliquity were negatively correlated with the recovery of lower limb movement [18, 19]. In addition, the pelvic obliquity was positively correlated with hip adduction and knee extension moment [19]. This might indicate that various deviations of the trunk may be compensatory. Therefore, it was a feasible method to reduce trunk compensation by improving lower limb movement in the gait of stoke.

Ankle foot orthosis (AFO) is widely used to improve stroke gait [20–22]. Among the various types of AFOs, passive AFOs are the most popular daily-wear device due to their durability and simplicity of design [22]. As the most commonly used non-articulated passive AFO, the rigid AFO (RAFO) could increase the ankle dorsiflexion to achieve heel strike at initial contact [23, 24] and promote foot clearance in swing phase [20, 25]. However, the RAFO restricted plantarflexion in loading response, and the dorsiflexion in late stance. In other words, the 2nd rocker function was negatively affected in gait. A hydraulic oil damper ankle joint in an AFO was developed based on the concept of 1st and 2nd rocker functions by Yamamoto et al. [26]. The AFO with an oil damper (AFO-OD) was an articulated AFO that transformed the ankle joint rotation into linear compression of the oil damper via a CAM mechanism, providing no dorsiflexion resistance. And the plantarflexion resistance should be adjusted appropriately for individuals and affect the progress of 1st and 2nd rockers in gait [27–37]. One study by Yamamoto et al. found that the AFO-OD promoted the gradual progression toward plantarflexion in loading response which promoted the lower limbs to be relatively extended alignment without causing the pelvis to tilt forward, indeed leading to a more upright posture of the thorax [38].

Plenty of studies had clarified the effect of the AFO-OD in view of low limb kinetics and kinematics, and only a few previous studies focused on the effect on the thorax and pelvis. Lan et al. found using AFOs improved the walking capacity by improving the stability and concordant of the trunk in hemiplegic patients [39]. Our previous studies also pointed out that AFO-OD had a positive effect on maintaining upright posture [38]. However, none of them conducted a systematic and comprehensive study of the thorax and pelvis movement. Therefore, we aimed to quantitatively analyze the effect of different designs of the AFOs (RAFO and AFO-OD) on the gait of patients with stroke from the thoracic and pelvic perspectives through clinical trials. The hypothesis of this study was that with the known effects of AFOs on the lower limb kinematic chain, patients using different types of AFOs would directly or indirectly affect the movement of thorax and pelvis in three-dimensional space.

## Results

**Fig. 1:**
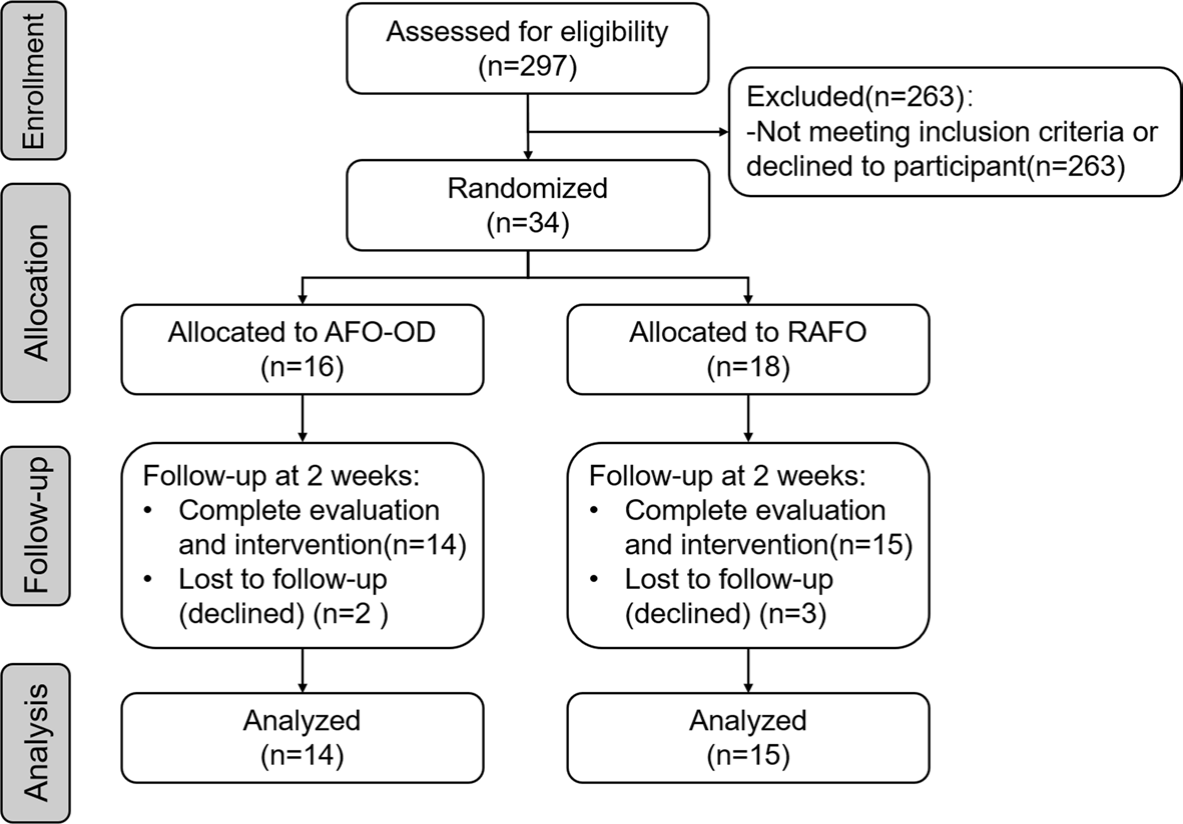
Flow diagram for patient’s selection process

A total of 29 patients completed the study and were analyzed (AFO-OD:14, RAFO:15) following the protocol of CONSORT 2010 **(**Fig. 1), 5 cases dropped out. There were no significant differences between the groups in age, body height, body weight, and time since onset, as shown in Table 1. There were no significant differences between the groups before training in any gait parameters.

**Table 1:** Profiles of participants with stroke

	AFO-OD (*n* = 14)	RAFO (*n* = 15)	*p*-value
Gender	Male: 12, female:2	Male: 12, female:3	
Age(years)	51.07 ± 12.55	50.07 ± 15.44	0.850
Body height(cm)	171.64 ± 7.97	168.33 ± 5.64	0.205
Body weight(kg)	72.64 ± 11.78	74.17 ± 13.04	0.744
BMI	24.52 ± 2.43	26.02 ± 3.38	0.183
Diagnosis	Cerebral hemorrhage:4	Cerebral hemorrhage:5	
	Cerebral infarction:10	Cerebral infarction:10	
Paretic side	Left:6, Right:8	Left:5, Right:10	
Time since onset(days)	154.64 ± 75.46	108.53 ± 45.84	0.055
Brunnstrom stage of lower extremities	III:5, IV:7, V:2	III:6, IV:7, V:2	
Fugl-Meyer balance	10.00(8–13)	10.00(8–12)	0.914
Functional ambulation category	II:4, III:6, IV:4,V:0	II:4, III:9, IV:2, V:0	

Fugl-Meyer Balance: median(range), others: mean ± standard deviation; AFO-OD: AFO with an oil damper; RAFO: rigid AFO; AFO: ankle foot orthosis

Table 2 shows the comparison results of 24 pelvic and thoracic angle parameters. Significant interaction effects (*p* < 0.05) were found in the pelvic tilt angle at contralateral foot off (CFO) and contralateral initial contact (CIC), pelvic rotation angle, and thoracic rotation angle at initial contact (IC). Within AFO-OD groups, the pelvic rotation angle at IC was significantly higher in the condition of wearing AFOs than not wearing AFOs (*p* = 0.012, *F* = 8.511). The main effects of AFO condition showed significantly more pelvic rotation to the non-paretic side at CFO (*p* = 0.003, *F* = 10.417) and less thoracic forward tilt at foot off (FO) (*p* = 0.015, *F* = 6.740) when patients wore AFOs. Inversely, the main effect of AFO type failed to reach statistical significance.

**Table 2:** Comparison of pelvic and thoracic kinematics in two AFO groups after gait training

		AFO-OD		RAFO		Interaction effect	Main effect		With vs Without		AFO-OD vs RAFO	
		Without	With	Without	With		AFO effect	AFO type	AFO-OD	RAFO	Without AFO	With AFO
Pelvic angle												
Initial contact	Forward/backward tilt (°)	**− 10.432**	**− 8.470**	**− 10.194**	**− 10.613**	0.075	0.240	0.698				
		7.315	6.133	6.591	6.957							
	Lateral obliquity (°)^a^	**2.336**	**1.465**	**3.358**	**2.459**				0.331	0.609	0.847	0.683
		3.360	4.448	2.794	4.770							
	Rotation (°)	**− 2.738**	**1.002**	**-0.030**	**− 0.133**	0.011*			0.012*	0.874	0.348	0.574
		6.348	6.220	5.679	6.032							
Contralateral foot off	Forward/backward tilt (°)	**− 12.838**	**− 10.865**	**− 13.749**	**− 14.377**	0.035*			0.072	0.336	0.686	0.183
		7.205	6.231	7.924	8.366							
	Lateral obliquity (°)^a^	**− 3.060**	**− 3.707**	**− 3.402**	**− 3.678**				0.158	0.256	0.621	0.451
		4.988	4.209	4.851	5.808							
	Rotation (°)	**3.579**	**6.701**	**3.262**	**4.684**	0.238	0.003**	0.582				
		5.639	6.478	5.347	6.286							
Contralateral initial contact"	Forward/backward tilt (°)	**− 13.908**	**− 11.924**	**− 14.968**	**− 16.295**	0.007**			0.653	0.104	0.061	0.057
		6.839	5.822	7.565	7.679							
	Lateral obliquity (°)^a^	**− 0.099**	**− 1.150**	**0.214**	**0.445**				0.158	0.609	0.847	0.621
		2.215	3.321	4.057	5.200							
	Rotation (°)	**1.653**	**2.231**	**0.962**	**0.384**	0.470	1.000	0.605				
		6.546	6.048	7.808	6.830							
Foot off	Forward/backward tilt (°)	**− 14.799**	**− 12.465**	**− 14.628**	**− 14.631**	0.072	0.073	0.678				
		6.830	5.820	6.794	6.930							
	Lateral obliquity (°)^a^	**2.566**	**1.208**	**1.671**	**1.874**				0.074	0.363	0.505	0.715
		4.258	4.889	3.919	3.911							
	Rotation (°)^a^	**− 3.486**	**− 3.613**	**− 2.565**	**− 4.739**				0.510	0.820	0.983	0.354
		10.492	9.443	13.095	11.360							
Thoracic angle												
Initial contact	Forward/backward tilt (°)^a^	**1.741**	**4.099**	**2.686**	**0.679**				0.048*	0.005**	0.331	0.123
		7.620	6.263	3.792	4.842							
	Lateral obliquity (°)^a^	**1.517**	**1.498**	**1.374**	**1.481**				0.925	1.000	0.847	1.000
		5.132	5.553	2.013	3.619							
	Rotation (°)	**− 3.579**	**− 1.202**	**− 2.675**	**− 3.696**	0.029*			0.077	0.241	0.899	0.088
		5.512	3.904	6.140	5.767							
Contralateral Foot off"	Forward/backward tilt (°)	**− 0.670**	**1.574**	**− 1.484**	**− 0.813**	0.336	0.081	0.312				
		4.240	3.693	5.636	4.919							
	Lateral obliquity (°)	**− 3.500**	**− 3.382**	**− 3.444**	**− 3.430**	0.876	0.842	0.997				
		1.987	2.928	3.552	3.046							
	Rotation (°)^a^	**2.160**	**4.616**	**1.629**	**1.183**				0.009**	0.233	0.451	0.085
		5.612	5.673	7.938	6.822							
Contralateral initial contact	Forward/backward tilt (°)	**− 2.269**	**0.335**	**− 2.926**	**− 2.521**	0.164	0.061	0.239				
		3.866	3.755	5.106	4.785							
	Lateral obliquity (°)	**− 2.911**	**− 2.802**	**− 2.510**	**− 2.557**	0.809	0.925	0.756				
		2.415	2.672	3.466	2.891							
	Rotation (°)^a^	**4.382**	**3.121**	**3.001**	**0.018**				0.245	0.910	0.290	0.146
		7.908	8.535	9.661	11.634							
Foot off	Forward/backward tilt (°)	**− 2.089**	**1.084**	**− 1.795**	**− 1.226**	0.082	0.015*	0.507				
		4.133	4.465	4.876	4.363							
	Lateral obliquity (°)^a^	**0.517**	**1.324**	**1.470**	**1.611**				0.470	0.140	0.505	0.652
		4.130	4.077	2.414	2.519							
	Rotation (°)^a^	**− 2.008**	**− 1.532**	**− 2.642**	**− 5.478**				0.300	0.496	0.983	0.070
		6.948	5.527	15.269	10.447							

Bold represents easier to distinguish them from SD or IQR

Upper column: mean or median; Lower column: SD or IQR

*AFO-OD*: AFO with an oil damper; *RAFO*: rigid AFO; *AFO*: ankle foot orthosis; Backward + ; non-paretic obliquity + ; non-paretic rotation +

^a^not normally distributed

^*^p < 0.05

^**^p < 0.01

We also found that when patients wore AFOs, the thoracic forward tilt angle at IC was significantly lower in the AFO-OD group (*p* = 0.048, *Z* = − 1.977) and higher in the RAFO group (*p* = 0.005, *Z* = − 2.783), while the thoracic rotation angle at CFO rotated more to the non-paretic side in the AFO-OD group (*p* = 0.009, *Z* = − 2.605).

The results for temporal and spatial factors, shank-to-vertical angle (SVA), and ground reaction forces are shown in Table 3. SVA angle was a parameter to evaluate AFO-Footwear Combination’s tuning, as it reflected concomitant changes in the lower limb angles and moments [40]. Significant interactions were found in the following parameters: the paretic to non-paretic step length, SVA forward inclination at FO, change of SVA in the stance, and SVA at CIC. Within AFO-OD groups, the following three parameters were significantly higher in the condition of wearing AFOs, compared to not wearing AFOs (*p* = 0.015, *F* = 7.927; *p* = 0.005, *F* = 11.576; *p* = 0.047, *F* = 4.808). Within RAFO groups, patients wearing AFOs showed significantly less value in the change of SVA in the stance and SVA forward inclination at CIC (*p* = 0.015, *F* = 7.654; *p* = 0.001, *F* = 17.327). The simple effect of the AFO type was also found in SVA at CFO (*p* = 0.023, *F* = 6.636), which indicated patients wearing AFO-OD showed more forward inclination than wearing RAFO. The significant AFO condition effects revealed that patients wearing AFOs had more SVA at IC (*p* = 0.006, *F* = 8.820) and more backward tilt SVA at CFO (*p* = 0.029, *F* = 5.312), whereas no significant main effects were found for any parameter of the AFO type.

**Table 3:** Comparison of temporal and spatial factors, SVA, and GRF in two AFO groups after gait training

	AFO-OD		RAFO		Interaction effect	Main effect		With vs without		AFO-OD vs RAFO	
	Without	With	Without	With		AFO effect	AFO type	AFO-OD	RAFO	Without AFO	With AFO
Temporal&spatial											
Velocity (m/s)^a^	**0.217**	**0.264**	**0.231**	**0.205**				0.026*	0.211	0.880	0.747
	0.219	0.191	0.119	0.144							
Step length (paretic to non-paretic) %height	**0.153**	**0.171**	**0.150**	**0.144**	0.009**			0.015*	0.343	0.916	0.262
	0.059	0.055	0.047	0.052							
Step length (non-paretic to paretic) %height^a^	**0.140**	**0.151**	**0.140**	**0.120**				0.035*	0.650	0.652	0.123
	0.108	0.098	0.040	0.064							
Cycle time (s)^a^	**1.983**	**2.018**	**1.947**	**1.833**				0.249	0.053	0.451	0.102
	1.178	1.100	0.627	0.507							
Loading response time (s)^a^	**0.547**	**0.623**	**0.575**	**0.570**				0.315	0.233	0.780	0.400
	0.850	0.637	0.263	0.217							
Single stance time (s)	**0.359**	**0.367**	**0.353**	**0.326**	0.140	0.420	0.473				
	0.097	0.099	0.076	0.096							
Preswing time (s)^a^	**0.431**	**0.433**	**0.485**	**0.517**				0.397	0.211	0.747	0.880
	0.491	0.421	0.188	0.239							
Swing time (s)	**0.602**	**0.592**	**0.546**	**0.499**	0.192	0.054	0.052				
	0.116	0.101	0.111	0.093							
SVA											
Initial contact (°)	**− 8.307**	**− 6.163**	**− 6.471**	**− 3.855**	0.771	0.006**	0.236				
	6.051	4.867	4.145	5.142							
End of loading response (°)	**3.550**	**2.960**	**4.125**	**1.745**	0.176	0.029*	0.893				
	7.647	6.699	6.157	5.780							
Start of preswing (°)	**9.157**	**9.982**	**10.103**	**5.157**	0.001**			0.364	0.001**	0.665	0.023*
	6.404	5.656	5.597	5.532							
Foot off (°)	**24.470**	**29.293**	**24.820**	**24.054**	0.005**			0.005**	0.528	0.919	0.155
	6.883	9.121	6.233	7.709							
Change in loading response (°)^a^	**10.429**	**7.798**	**7.821**	**5.177**				0.004**	0.001**	0.425	0.310
	9.372	10.631	8.045	8.407							
Change in stance (°)	**32.777**	**35.456**	**31.291**	**27.910**	0.002**			0.047*	0.015*	0.627	0.120
	11.111	12.379	8.191	10.378							
Change in single stance (°)^a^	**4.984**	**5.799**	**5.436**	**2.433**				0.048*	0.001**	0.880	0.037*
	4.915	6.705	4.508	5.867							
GRF											
Peak posterior (N/kg)^a^	**− 0.488**	**− 0.432**	**− 0.619**	**− 0.654**				0.363	0.281	0.123	0.270
	0.281	0.220	0.205	0.294							
Peak anterior (N/kg)	**0.392**	**0.445**	**0.334**	**0.336**	0.192	0.148	0.174				
	0.129	0.182	0.180	0.178							

Bold represents easier to distinguish them from SD or IQR

Upper column: mean or median; Lower column: SD or IQR

*AFO-OD* AFO with an oil damper; *RAFO* rigid AFO; *AFO* ankle foot orthosis; *SVA* Shank-to-vertical angle; *GRF* ground reaction force; *SVA* forward inclination + ; *GRF* forward +

^a^not normally distributed

^*^p < 0.05

^**^p < 0.01

Compared to patients without AFOs, it is shown that change of SVA during loading response was significantly lower in both groups with AFOs (AFO-OD: *p* = 0.004, *Z* = − 2.856; RAFO: *p* = 0.001, *Z* = − 3.237). Within AFO-OD groups, velocity, non-paretic to paretic step length, and change of SVA in single stance were significantly higher (*p* = 0.026, *Z* = − 2.229; *p* = 0.035, *Z* = − 2.103; *p* = 0.048, *Z* = − 1.977) when patients wore AFOs. Within RAFO groups, change of SVA in single stance was significantly lower (*p* = 0.001, Z = − 3.408) when patients wore AFOs. Additionally, patients with AFO-OD showed more change of SVA in single stance than with RAFO (*p* = 0.037, *U* = 57.000).

The results for ankle, knee, and hip joint kinematics of paretic side are shown in Additional file 1.

## Relevance

**Fig.2:**
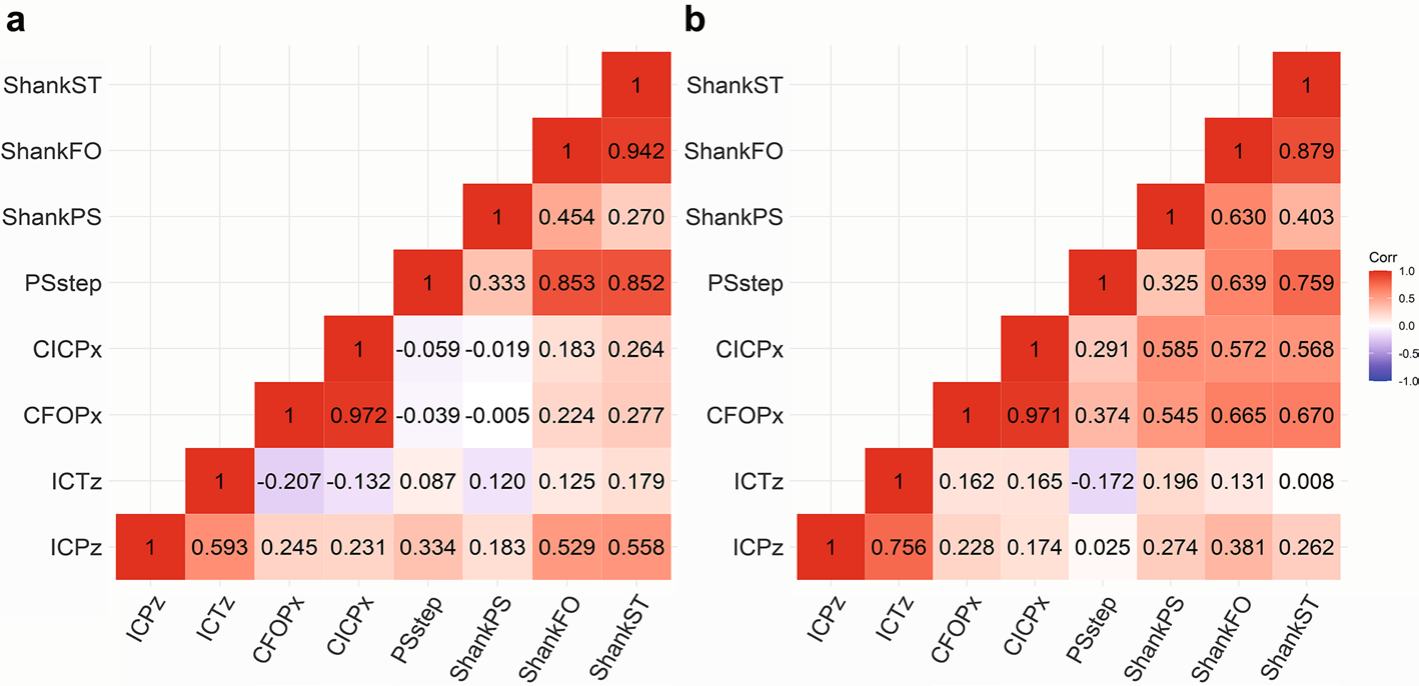
Correlation coefficient between parameters: **a** AFO-OD group; **b** RAFO group. ICPz: pelvic rotation angle at initial contact; ICTz: thoracic rotation angle at initial contact; CFOPx: pelvic tilt angle at contralateral foot off; CICPx: pelvic tilt angle at contralateral initial contact; PSstep: paretic to non-paretic step length; ShankCIC: shank vertical angle at contralateral initial contact; ShankFO: shank vertical angle at foot off; ShankST: the change of shank vertical angle in the stance

The correlation coefficient between parameters that showed interactions within each group wearing AFOs in this study was calculated and is shown in Fig. 2. The change of SVA was found to correlate with pelvic rotation at initial contact in the AFO-OD group and with a pelvic tilt at the contralateral foot off and contralateral initial contact in the RAFO group.

SVA was positively correlated with pelvic tilt at contralateral foot off (*r* = 0.670, *p* = 0.006) and at contralateral initial contact (*r* = 0.568, *p* = 0.027) in RAFO group, but not in the AFO-OD group. But SVA was positively correlated with pelvic rotation in the AFO-OD group (*r* = 0.558, *p* = 0.038), not in the RAFO group. What is more, SVA was correlated with paretic step length in both groups. Pelvic rotation was correlated with thoracic rotation in both groups (AFO-OD group: *r* = 0.593, *p* = 0.025; RAFO group: *r* = 0.756, *p* = 0.001).

## Discussion

In the current study, we evaluated the effect of two AFOs, namely, AFO-OD and RAFO on the pelvic and thoracic movements in the gait of stroke patients after two weeks of training. Our results proved the hypothesis that there were direct effects on the thorax and pelvis movement using different AFOs in patients with stroke, and the effects were different.

When the patient wore the AFO-OD, the pelvis rotated more to the non-paretic side at initial contact, which was followed by an increase in step length, speed, and change of SVA in stance, compared to not wearing the AFO. Some studies also showed that pelvic rotation anteriorly translated the hip, contributing to increased stride length and higher speed [41, 42]. We supposed that a larger change of SVA might represent better comfort, considering that SVA peaked when the patient felt most comfortable [33]. Additionally, the pelvic rotation at initial contact was positively correlated with the change of the SVA in stance. Meanwhile, the knee joint did not show excessive flexion at initial contact and still approached the 5° flexion (5.88 ± 7.14° at initial contact wearing AFO-OD, see Additional file 1) and was not a compensation for limited hip flexion, which was shown in normal gait [43, 44]. This had a positive effect on gait. On the other hand, resistive plantarflexion movement in loading response was enabled in the AFO-OD, the loading response occurred gradually with the assistant of the plantarflexion resistance generated by the oil damper, and the knee joint was not pushed excessively flexed [35]. The improvement of the pelvic rotation based on a relatively upright posture of lower limbs and synthesized with gait parameters demonstrated the positive value of AFO-OD for gait improvement in stroke patients.

In the RAFO group, the patient walking with AFO instead led to a significantly decreased change of SVA in stance (with AFO:27.91 ± 10.38°, without AFO: 31.29 ± 8.19°), which was even less than when the patient walked without AFO in the AFO-OD group (with AFO: 35.46 ± 12.38°, without AFO: 32.78 ± 11.11°). RAFO generated overlarge plantar flexion and dorsiflexion resistance, stopping ankle dorsiflexion and limiting shank forward inclination in the paretic stance. And the restriction of shank forward progression in the RAFO group also notably provided a control effect on the knee joint and prevented excessive knee flexion phenomenon in late stance. We even observed knee hyperextension (− 1.97°), which could impair walking speed, reduce gait symmetry, decrease gait efficiency, increase use of energy during walking, and might be associated with knee pain [46]. Moreover, the reduced change of SVA in stance indicated the restriction to the progression of the 2nd rocker function in the RAFO group, which was significantly correlated with the pelvic forward tilt at CFO in the RAFO group (*r* = 0.670, *p* = 0.006), as pelvic and thoracic forward tilt was generally acknowledged to be a common characterized phenomenon in the gait of stroke [47]. However, only a limited number of studies concluded the effect of various types of AFOs on the pelvis and thorax prior to the current study [38].

Another notable finding in this study was that the thoracic rotation to non-paretic side at IC and CFO increased in the AFO-OD group and was positively correlated with the pelvic rotation at IC in both groups. The thorax and pelvis rotated synchronously in the same direction, which is called an in-phase rotation. In normal gait, the thorax and pelvis rotated more anti-phase (opposite direction) [42], whereas in stroke gait, they rotated more in-phase, higher (more anti-phase) in stroke patients with lower gait impairments indicating more dissociation of the thoracic and pelvic segments [15]. Increased pelvic rotation was considered to be a positive sign, while the increased in-phase rotation of the thorax might be attributed to the lack of coordination of impaired trunk movement of stroke patients. In patients with stroke, both sides of the trunk were impaired and characterized by diminished synchronization and lower activity levels of the trunk muscular system [45], manifesting as the weakness of dissociation and the lack of coordination of the thoracic and pelvic movements. Although after two weeks of AFO adaptive training, the pelvic control of patients with stroke showed relatively optimum performance with the assistance of plantarflexion resistance in the AFO-OD group, trunk impairment affected pelvic and thoracic separation movements, which may require longer rehabilitation cycles. This also prompted us to consider recommending rehabilitation therapists to conduct targeted training for patients in the gait training of AFO. The arm swings forward as the contralateral leg steps forward in normal gait, and vice versa [15]. According to the recovery principle of stroke gait, we propose that ideas and thinking about whether it is possible to adapt to the effect of plantarflexion in the orthosis training of hemiplegic patients, special training, and guidance could be carried out for rotating dissociation movement and coordination control of the trunk, for example, prompt the patient to increase the swing of the contralateral upper limb to promote the inverted rotation of thorax versus pelvis.

The AFO-OD with plantarflexion resistance enabled the gradual movement to plantar flexion in loading response. With the kinematic chain of the lower limb, relatively upright alignment of the lower limb and improved pelvic rotational movement resulted in the improvement in velocity, step length, and shank progression, which meant better performance, although there was still in-phase rotation of the pelvis and thorax. The RAFO with plantarflexion and dorsiflexion stop decreased SVA progression in stance, affecting the forward tilt of the pelvis, and might result in knee hyperextension in late stance. This study suggested that clinicians should give more consideration to the effects of different types of AFOs on trunk posture in clinical gait training for stroke patients, and in the future, we would conduct more studies on AFOs based on this research and explore the correlation between lower limb biomechanics and trunk biomechanics in stroke gait.

This study has some limitations. Firstly, the resistive moment of the AFO-OD was the same in this study, but it should probably be adjusted for each patient. The trim line and elasticity might affect the fixed resistance generated by the RAFO, but it could not be assessed in this study. Secondly, this study included a relatively wide range of patients in Brunnstrom stage III–V. Although the degree of spasticity of the patient was controlled by MAS, there might be potential differences in dissociation movements and spasticity of lower limbs among different Brunnstrom stages. Therefore, the gait of patients should be analyzed in each Brunnstrom stage in future studies.

## Conclusion

This randomized controlled trial assessed the effect of different types of AFOs on gait with stroke by comparing the effects of a rigid AFO, which relatively stopped both dorsiflexion and plantarflexion, and the AFO-OD which generated plantarflexion and enabled free dorsiflexion. The findings in 29 patients with stroke showed the paretic step length, pelvic and thoracic rotation at initial contact, and the change of SVA in single stance improved more significantly in participants who underwent gait training using an AFO-OD than in those using a RAFO. Pelvic rotation at initial contact correlated with the change of SVA in stance and thoracic rotation. Pelvic rotation and lower limb kinematics exhibited significant improvements with AFO-OD, reflecting more desirable gait performance. On the other hand, the increase in the thoracic in-phase rotation might expose the effect of insufficient trunk control and dissociation movement.

## Methods

### Participants

A total of 34 patients with subacute stroke (more than 14 and less than 180 days after onset) who were hospitalized for rehabilitation treatment in a rehabilitation facility in China from June 2020 to August 2021 participated in this study. The following inclusion criteria and exclusion criteria were applied. The inclusion criteria were as follows: Brunnstrom stage of the lower limb over grade II; age within 18–80 years old; and ability to walk safely on level ground using any type of AFOs, using canes if necessary. The exclusion criteria included spasticity grade over 2 on the Modified Ashworth Scale, musculoskeletal or cognitive problems, and pregnancy.

All participants underwent gait training under the supervision of physiotherapists. None had previously used an AFO, and all were enrolled in the study when they began walking 8 m under supervision. All procedures were approved by the local ethics committee of the International University of Health & Welfare (No.19 Io-144–2) and China Rehabilitation Research Center (2019–116-2). Informed consent was obtained from all participants before they participated in this study.

### AFOs and equipment

**Fig. 3:**
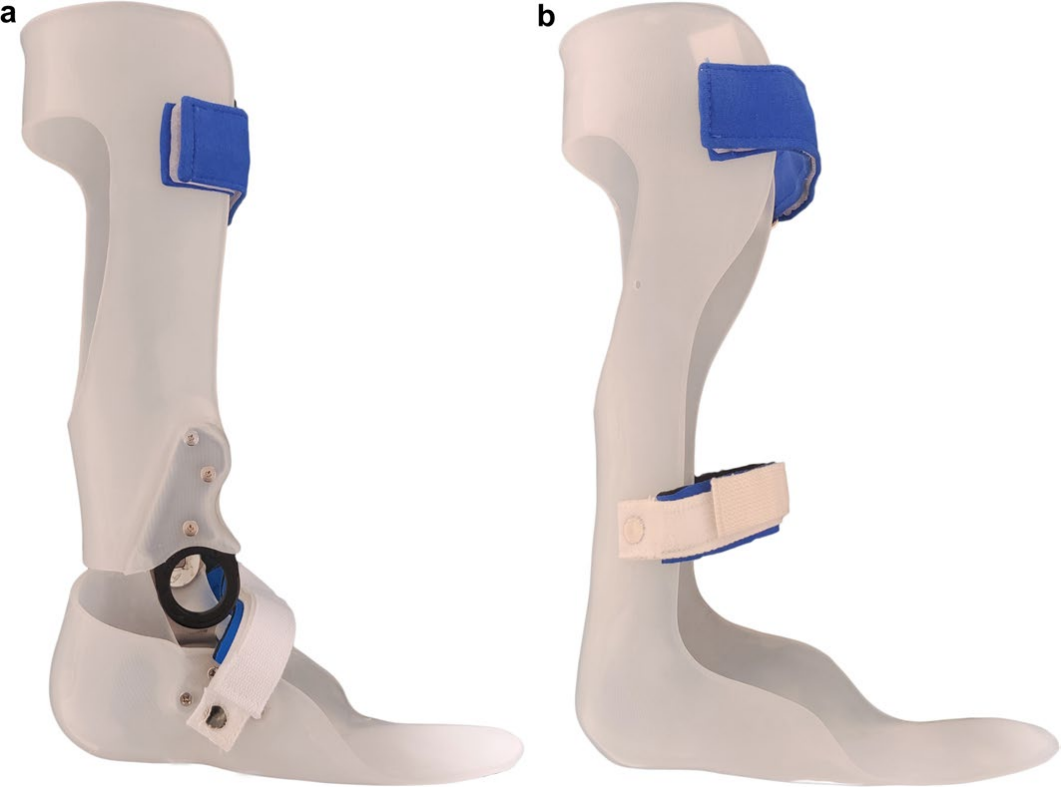
AFOs used in this study. **a** AFO-OD: AFO with an oil damper; **b** RAFO: plastic customized rigid AFO

**Fig. 4:**
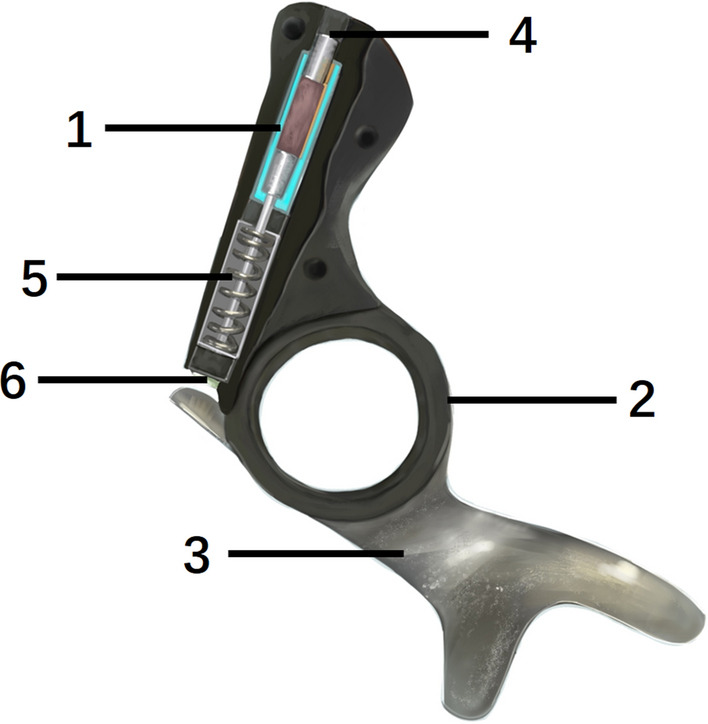
A schematic design of the AFO with oil damper unit. The oil damper unit consists of a hydraulic cylinder(1), a ring portion metal plate (2), and a metal plate (3). An adjustment screw (4) controls the flow rate of the oil by varying the orifice diameter; the smaller the orifice diameter, the lower the flow rate, allowing for greater resistance to plantarflexion of the ankle joint at heel strike. A spring (5) assists with dorsiflexion. The rod cap (6) is used to set the initial angle of the ankle joint

The two types of AFOs shown in Fig. 3 were used in this study. The customized AFO-OD (Gait Solution, Kawamura-gishi, Osaka, Japan) **(**Fig. 3a**)** had a mechanical ankle joint with an oil damper, providing a free dorsiflexion movement from 0 to 8°. Figure 4 shows the schematic design of the oil damper unit. The plantarflexion resistance generated by the oil damper could be adjusted in 5–14 Nm at 10° of plantarflexion from 1 (flexible) to 4 (rigid). In this research, the magnitude of the oil damper was set to moderate magnitude OD2.5 according to the result of prior studies [38, 48]. The customized plastic RAFO **(**Fig. 3b**)** had no mechanical joints, and ankle trimming lines covered the malleoli.

The initial ankle joints of both AFOs were set to a neutral position. All AFOs in two groups were customized by three orthotists in the prosthetic and orthotic department of the facility. Eight oil damper joints were prepared (4 right and 4 left) for the AFO-OD group. The AFOs were fitted well to ensure suitability.

Gait was measured by a three-dimensional (3D) motion capture system with 6 motion capture cameras (Qualisys AB, Sweden) and 2 force plates (Bertec Corp, USA), which were placed diagonally adjacent to each other in the middle of the eight-meter walkway. Thirty-seven infrared reflective markers were positioned at specified landmarks of participants according to Plug-In-Gait Marker Placement. Markers were set on the lower limbs (both metatarsophalangeal joints, heels, ankles, shanks, knees, thighs, and hips) and the upper limbs (both wrists, elbows, and shoulders). Markers were also placed on the thorax (the spinous processes of the 7th cervical vertebra, the 7th thoracic vertebrae, the xiphoid process of the sternum, and the jugular notch where the clavicles meet the sternum) and on the pelvis (both anterior superior iliac spines and the posterior superior iliac spines). The trajectories of markers and the ground reaction force data were sampled at the frequency of 200 Hz and 1000 Hz, respectively.

### Study protocol

First, shod gait without AFO was measured at each participant’s self-selected walking speed in an 8-m walkway. Each participant was required to land their hemiplegic lateral foot exactly intact on one of the force platforms during one test and repeat three times. We divided the gait cycle according to the ground reaction force and analyzed this gait cycle.

Next, the participants were randomly allocated to an AFO-OD group or a RAFO group in order of participation. Then, the participants started gait training sessions that were performed for 1 h daily over 2 weeks under the supervision of physiotherapists. The training sessions were the same for both groups and included the simulated practical walk and general exercises, such as range of motion exercises, balance training, muscle training, and step training. At last, after 2 weeks, gait was measured with and without allocated AFOs separately using the same method.

### Data processing

Marker trajectories and force plate data were low-pass filtered by a second-order Butterworth filter with cutoffs of 6 and 18 Hz, respectively. The gait cycle was defined as the loading response, single stance, preswing, and swing phase of the paretic limb. These phases were distinguished by the vertical component of the ground reaction force (GRF) with a force threshold of 10 N for heel contact and toe off the ground. Joint kinematics and kinetics were calculated using an inverse dynamic model. The pelvic and thoracic angles were calculated as the Euler angles at four moments in three planes, respectively. The four moments included IC, CFO, CIC, and FO. Initial contact of the paretic side was defined as IC, and the IC of the non-paretic side was defined as the CIC. The angle of inclination of the shank segment to the vertical (shank-to-vertical angle, SVA) in the sagittal plane was calculated given its importance in assessing the effect of AFOs on gait[33]. The GRF was normalized by body weight. Step length was normalized by each participant’s body height.

A total of 24 pelvic and thoracic parameters, 17 temporal and spatial factors, SVA, and ground reaction forces, and 16 lower extremity joint kinematics were calculated in this study. Visual 3D software version 2020.11.2 (C-Motion Inc., Kingston, ON, Canada) was used in all the post-data processing.

### Statistical analysis

All gait parameters were calculated as the average of three gait cycles for each condition, with/without an AFO before training and after training. All data were checked for normality distribution by the Shapiro–Wilk test.

First, we compared the consistency of the baseline data, including the first shod gait data without AFO and general information. An independent t-test was performed for normally distributed data and a Mann–Whitney *U* test was performed for the data which were not normally distributed in this procedure.

To know the effect of different types of AFOs after the gait training, two-way repeated ANOVA was performed for normally distributed data, with paired factors (condition, with and without AFOs) and an unpaired factor (type of AFOs). If no interaction was found, the main effects of the two factors were assessed. In cases of interaction, a comparison was made between conditions in each AFO group using one-way ANOVA. For the data that were not normally distributed, the effect of the conditions was compared using the Wilcoxon signed-rank test and the effect of the types of AFOs was examined using the Mann–Whitney *U* test.

Finally, the correlation analysis of the parameters showing significant interaction was done for each group. The level of significance was set at *p*-values of less than 0.05. All statistical analysis were performed using SPSS for Windows version 23 (IBM Corp., Armonk, NY).

## Supplementary Information

**Additional file 1: **Table of the comparison of lower limb joint kinematics between and within two AFO groups after gait training.

## Data Availability

The raw data were generated at China Rehabilitation Research Center. Derived data supporting the findings of this study are available from the author H.L. on request.

## Abbreviations

AFO: Ankle foot orthosis
AFO-OD: AFO with an oil damper
RAFO: Rigid ankle foot orthosis
IC: Initial contact
CFO: Contralateral foot off
CIC: Contralateral initial contact
FO: Foot off
SVA: Shank-to-vertical angle
